# *Tuber aztecorum* sp. nov., a truffle species from Mexico belonging to the Maculatum clade (Tuberaceae, Pezizales)

**DOI:** 10.3897/mycokeys.30.22887

**Published:** 2018-02-28

**Authors:** Gonzalo Guevara-Guerrero, Gregory Bonito, Matthew E. Smith, Rosanne Healy, Arthur C. Grupe II, Efrén Cázares, Michael A. Castellano, James M. Trappe

**Affiliations:** 1 Instituto Tecnológico de Cd. Victoria, Av. Portes Gil 1301 Pte. C.P. 87010, Cd. Victoria Tam. México; 2 Department of Plant, Soil and Microbial Sciences, Michigan State University, East Lansing, Michigan, 48825, USA; 3 Department of Plant Pathology, University of Florida, Gainesville, Florida 32611, USA; 4 US Department of Agriculture, Forest Service, Northern Research Station, 3200 Jefferson Way, Corvallis, Oregon, 97331, USA

**Keywords:** Taxonomy, systematics, phylogeny, hypogeous fungi, cryptic species

## Abstract

A new species of truffle, *T. aztecorum*, is described from central Mexico. *Tuber aztecorum* can be distinguished from other related *Tuber* species synoptically by a combination of morphological features including ascospore size, pellis cells with irregular thickness, cystidia, ascoma colour and associated host (*Abies religiosa* an endemic *Abies* species from central Mexico); sequence variation on the ITS rDNA also distinguishes *T. aztecorum* from related species. A phylogenetic analysis of the ITS rDNA demonstrates that *T. aztecorum* belongs to the Maculatum clade and is unique from other similar small, white-cream coloured *Tuber* species distributed in north-eastern Mexico such as *T. castilloi* and *T. guevarai*.

## Introduction

*Tuber* is one of the most important edible truffle genera in the world due to its economic importance and ecological role in forest ecosystems. *Tuber* spp. are known as ‘true truffles’ and their fruiting bodies are edible and highly valued. Ecologically, *Tuber* spp. form symbiotic ectomycorrhizal associations with gymnosperm and angiosperm trees and also orchids (Riousset et al. 2001, Wurzburger et al. 2001, Bidartondo et al. 2004, Walker et al. 2005, Mello et al. 2006, Trappe et al. 2009, Martin and Bonito 2012, Morcillo et al. 2015). In addition, *Tuber* spp. are consumed for nutrition by many kinds of invertebrates and vertebrates including primates (McGraw et al. 2002, Hanson et al. 2003, Hochberg et al. 2003, Maser et al. 2008, Beaune et al. 2013). A few *Tuber* species are now cultivated worldwide, including *Tuber melanosporum*, *T. aestivum* and *T. borchii* (Martin and Bonito 2012).

The Puberulum and Maculatum clades within the genus *Tuber* are two of the most species diverse and geographically widely dispersed of the eleven recognised clades. More recently, the related Latisporum clade was described from Asia, where the species are endemic (Fan et al. 2016).*Tuber* species in these three clades are often pale in colour and typically small in size (Trappe et al. 2009, Bonito et al. 2010a, 2013, Guevara et al. 2013b, Payen et al. 2014). Recent molecular analyses of *Tuber* spp. from northern and central Mexico and USA have shown that *Tuber* species are genetically unique compared to their European and Asian counterparts (Bonito et al. 2009, 2010a, Lancellotti et al. 2016). Many *Tuber* species belonging to these clades have been formally named recently. For example, five species within the *T. separans* complex of the Puberulum clade were described from Mexico. *Tuber bonitoi*, a large truffle (approx. 5 cm) found recently in Mexico, is morphologically similar to *T. borchii*. It was found associated with *Pinus hartwegi* and *Abies religiosa*. *Tuber brunneum*, a smaller, brownish truffle from central Mexico, was associated with *Quercus magnolifolia* as was *T. pseudoseparans* and *T. tequilanum*. *Tuber guzmanii* and *T. separans* are also found in Mexico and belong to the Puberulum clade (Guevara et al. 2015). Other *Tuber* species belonging to the Maculatum clade are known from north-eastern and central Mexico including *Tuber castilloi*, *T. gardneri*, *T. guevarai*, *T. maculatum*, *T. mexiusanum* and *T. miquihuanense* (Cázares et al. 1992, Guevara et al. 2008, 2013a,b, 2015). In addition, new findings on asexual anamorphic states have been discovered for some North American *Tuber* species, however the role of these structures is still unknown (Urban et al. 2004, Ouanphanivanh et al. 2008, Healy et al. 2013).

Studies on *Tuber* species from Mexico are still scarce. In this work, a morphological and molecular analysis was performed on recent *Tuber* collections. The authors report on a new taxon, which is described here as *T. aztecorum*. Phylogenetically, *T. aztecorum* is within the Maculatum clade, a group of small to medium sized, white truffles. It is associated with *Abies religiosa*, an endemic *Abies* species from central Mexico. *Tuber aztecorum* can be differentiated from related taxa by its morphology, ecology, biogeography and nuclear ITS ribosomal DNA. This research contributes to the knowledge of *Tuber* biodiversity and ecology in North America.

## Materials and methods

### Sampling and morphological characterisation

*Tuber* fruiting bodies were collected from central México and preserved following recommendations of Harkness (1899) and Castellano et al. (1989). Duplicate splits of sample collections are deposited in the herbaria José Castillo Tovar (ITCV), Oregon State University (OSC), Michigan State University (MSU) and Florida University (FLAS). Previously accessioned herbarium specimens of *Tuber*, including type collections from OSC and ITCV, were also examined during this study.

Morphological data were obtained by the methods of Castellano et al. (1989), Gilkey (1916, 1939) and Pegler et al. (1993). Examined characters included ascoma (fruiting body) size, surface texture and colour, peridial structure; spore length and width (excluding ornamentation), length/width ratio (Q), shape, wall thickness, number of reticular meshes, height of the meshes, colour and ascus size, shape, wall thickness and number of spores/ascus. Hand-cut sections were mounted in 5% KOH and Melzer’s reagent for light microscopy. Spore measurements of *Tuber* spp. in KOH compared to those in water showed no KOH effect (J. Trappe, unpublished data). Microscopic structures were measured and photographed under a light microscope and stereo microscope.

### DNA sequencing and phylogenetic analyses

Molecular protocols follow those of Guevara et al. (2008). DNA was extracted from truffle fruiting bodies with the chloroform extraction technique using CTAB 2X DNA extraction buffer. The ITS region was amplified with the primer pair ITS1f-ITS4 (Gardes and Bruns 1993, White et al. 1990). PCR products were cleaned enzymatically with antarctic phosphatase and endonuclease digestion (New England Biolabs, Ipswich MA). Sanger sequencing was performed by Big Dye chemistry v3.1 (Applied Biosystems, Foster City, CA) with the forward primer ITS1f and reverse primers ITS4. DNA sequences were determined on an ABI 3700 capillary sequencer (Applied Biosystems, Foster City CA). DNA sequences were viewed and manually edited in Sequencher 4.0 (Gene Codes, Ann Arbor, MI). Sequences were aligned with MUSCLE (Edgar 2004). Alignments were manually checked and ambiguous regions were excluded in Mesquite 2.5 (Maddison and Maddison 2009).

Phylogenetic analyses were conducted with maximum likelihood (ML) in PAUP* (Swofford 2002). The best fit nucleotide substitution model (GTR+G+I) was based on the Akaike information criterion and was implemented in PAUP* 4d106 (Swofford 2002). ML bootstrap support based on 1000 replicates was assessed with RAxML (Pattengale et al. 2009, Stamatakis et al. 2008, Stamatakis 2006) and executed on the CIPRES Science Gateway (Miller et al. 2010). Phylogenetic trees were rooted with species belonging to the Latisporum clade. Sequences produced in this study are deposited in GenBank under accession numbers KY271791 and KY271790, Table 1.

**Table 1.** List of *Tuber* species, GenBank accession numbers and reference for the ITS sequences used in the phylogenetic analysis. The sequences of the new taxon are in bold.

**Taxon**	**GenBank**	**Reference**
*Tuber alboumbilicum* Y. Wang & Shu H. Li	KJ742702	Li et al. 2014
*T. anniae* W. Colgan & Trappe	NR119860	Bonito et al. 2010a
T. aff. asa Tul. & C. Tul.	HM485341	Bonito et al. 2010a
***T. aztecorum*** Guevara, Bonito & Smith	**KY271790, KY271791**	This paper
*T. beyerlei* Trappe, Bonito & G. Guevara	NR119866	Bonito et al. 2010a
*T. bomiense* K. M. Su & W. P. Xiong	KC517481	NCBI
*T. bonitoi* G. Guevara & Trappe	JT32421, KC152256,	Guevara et al. 2015
*T. borchii* Vittad.	HM485342	Bonito et al. 2010a
*T. brunneum* G. Guevara, Bonito & Trappe	JT33830, JT33837	Guevara et al. 2015
*T. californicum* Harkn.	HM485346	Bonito et al. 2010a
*T. castilloi* G. Guevara, Bonito & Trappe	NR119865	Guevara et al. 2013b
*T. cistophilum* P. Alvarado, G. Moreno, Manjón, Gelpi & J. Muñoz	JN392231	Alvarado et al. 2012
*T. dryophilum* Tul. & Tul.	HM485354	Bonito et al. 2010a
*T. foetidum* Vittad.	JQ288907	NCBI
*T. guevarai* Bonito & Trappe	JF419305	Guevara et al. 2013b
*T. huizeanum* L. Fan & C. L. Hou	JQ910651	Fan et al. 2012c
*T. latisporum* Juan Chen & P.G. Liu	NR119620	Chen and Liu 2007
*T. lauryi* Trappe, Bonito & G. Guevara	NR119862	Bonito et al. 2010a
*T. lijiangense* L. Fan & J.Z. Cao	GQ217541	Chen and Liu 2007
*T. linsdalei* Gilkey	HM485370	Bonito et al. 2010a
*T. liyuanum* L. Fan & J.Z. Cao	NR111717	Fan and Cao 2012a
*T. maculatum* Vitadd.	KJ524540	Hilszczanska et al. 2014
*T. mexiusanum* G. Guevara, Bonito & Cázares	NR119867	Guevara et al. 2013b
*T. microsphaerosporum* L. Fan & Y. Li	KF805726	Fan and Yue 2013
*T. microverrucosum* L. Fan & C.L. Hou	JN870099	Fan et al. 2012c
*T. miquihuanense* G. Guevara, Bonito & Cázares	NR119868	Guevara et al. 2013b
*T. panzhihuanense* X.J. Deng & Y. Wang	JQ978644	Deng et al. 2013
*T. pseudoseparans* G. Guevara, Bonito & Trappe	JT33778, JT33774 (KT897480)	Guevara et al. 2015
*T. pseudogamagnatum* L. Fan	NR111718	Fan and Cao 2012a
*T. pseudosphaerosporum* L. Fan	KF744063	Fan and Yue 2013
*T. rapaeodorum* Tul. & C. Tul.	DQ011849	NCBI
*T. separans* Gilkey	HM485385	Bonito et al. 2010a
*T. shearii* Harkn.	HM485389	Bonito et al. 2010a
*T. sinosphaerosporum* L. Fan, J.Z. Cao & Yu Li	JX092086	Fan and Yue 2013
*T. sphaerosporum* Gilkey	HM485390	Fan and Yue 2013
*T. tequilanum* G. Guevara, Bonito & Trappe	JT33755, JT33790 (KT897482)	Guevara et al. 2015
*T. vesicoperidium* L. Fan	JQ690071	Fan et al. 2012b
*T. walkeri* Healy, Bonito & G. Guevara	JF419265	Guevara et al. 2013b
*T. zhongdianense* X.Y. He, Hai M. Li & Y. Wang	DQ898187	Chen and Liu 2007
*Tuber* sp.	AB553464	Kinoshita et al. 2011
*Tuber* sp. 14	GQ221447	NCBI
*Tuber* sp. 36	JF419253, JF419256	Guevara et al. 2013b
*Tuber* sp. 47	HM485416	Bonito et al. 2010a
*EcM Salix humboldtiana* Willd.	KF742730	Berch and Bonito (2016)
*EcMCU046*	KJ595014	NCBI

## Results

### Molecular analyses

**Figure 1.**
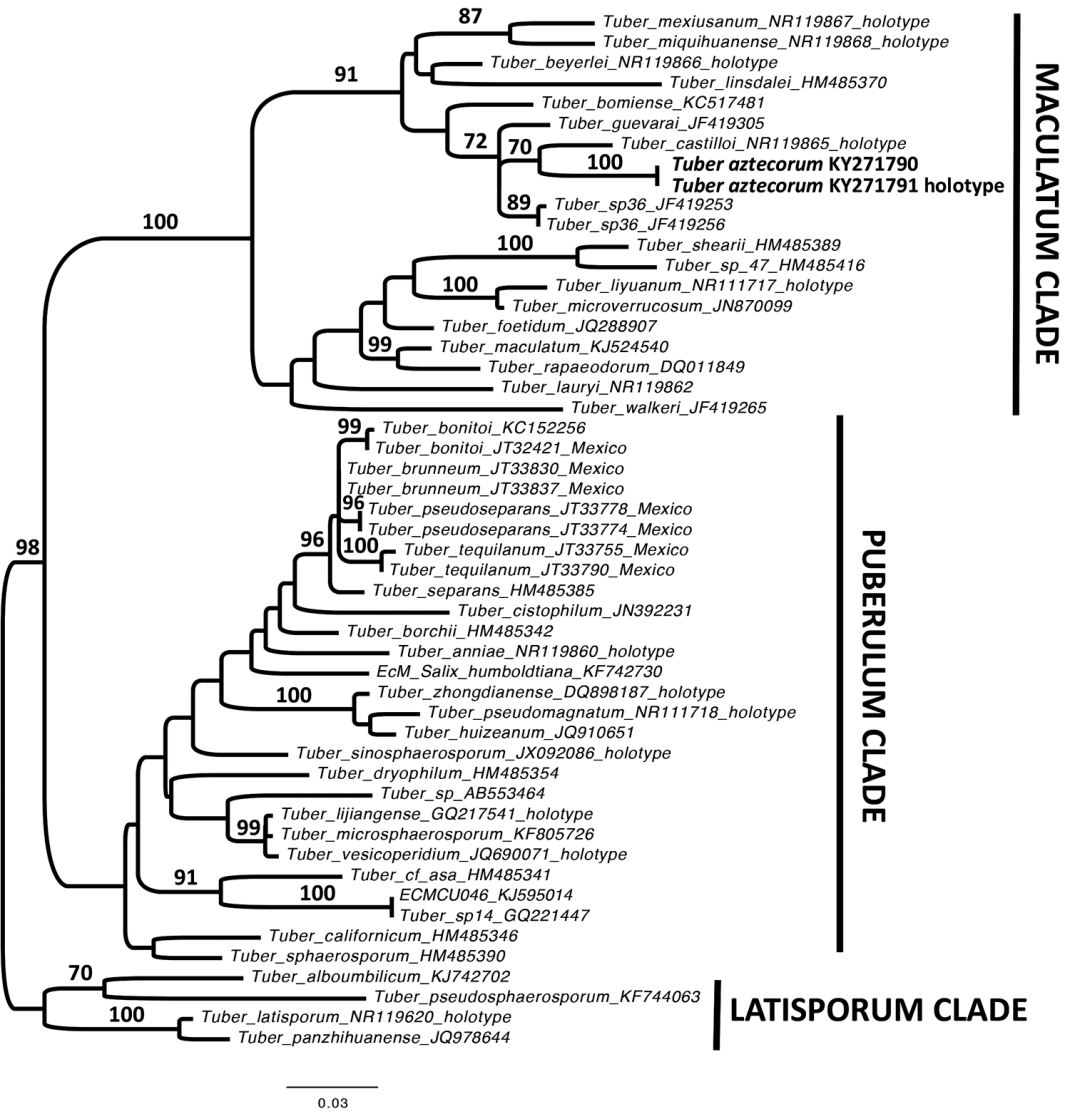
Phylogenetic tree inferred under the maximum-likelihood (ML) criterion from the ITS rDNA alignment corresponding to the *Tuber* dataset. The tree was rooted using midpoint rooting. Numbers on the branches represent support values from 1,000 ML bootstrap replicates. The branches are scaled in terms of the expected number of substitutions per site. The phylogeny is rooted with species belonging to the Latisporum clade. Accession numbers in the sequence labels indicate sequences from Genbank.

A total of 51 taxa including holotypes were analysed (Table 1). As previous studies have shown, the Maculatum clade was distinct from the Puberulum and Latisporum clades in ML and Bayesian Inference analyses (Fig. 1). The designation of *Tuber aztecorum* as a new species is supported by ITS rDNA analysis, morphological characters and ecology.

### Taxonomy

#### Tuber aztecorum

Taxon classificationFungiPezizalesTuberaceae

Guevara, Bonito & Smith sp. nov.

MB819367

Figs 1 , 2A–I

##### Type.

MEXICO. *State of Mexico*, Toluca-Temascaltepec road, La Puerta, Parque Nacional Nevado de Toluca, 29 July 2010, Guevara 993 (ITCV [José Castillo Tovar herbarium] – holotype, MSU and FLAS – isotypes), GB KY271791.

##### Diagnosis.

*Tuber aztecorum* is a sister species to *T. castilloi*, but *T. castilloi* differs by having larger spores, 27–63 × 20–40 µm, is without an irregular thickness to the cell wall on peridial hyphae and is associated mainly with *Quercus* spp. Also resembles *T. guevarai* but *T. guevarai* has narrow spores that are 18–55 × 16–42 µm and cream-yellow fruiting bodies and rDNA variation.

##### Etymology.

“*aztecorum*” in reference to the ancient Aztec civilisation of Mexico.

##### Description.

***Ascomata*** 5–23 × 4–16 × 3–11 mm, subglobose, irregular, lobate or globose, light to orange brown or reddish-brown changing to dark brown when handled, finely verrucose or granulose, with 5–8 verrucae in 1 mm, solid, brittle, surface dry, base sessile. Peridium in cross-section undetachable <.5 mm wide, with one or several basal white to cream furrows or depressions that merge into veins. 5% KOH negative. Gleba marbled, white to greyish, white veins, some veins ending in the peridium. Odour fungoid to raw potato-like, taste not recorded.

***Peridium*** 110–350 µm thick. Outer layer (epicutis) a pseudoparenchyma 62–250 µm thick, of hyphae 5–30 µm diam., versiform, angular or isodiametric, in some areas hyphae arranged perpendicular to the epicutis, hyaline to reddish-brown in mass in KOH, thick-walled (2 µm), without intracellular content. Surface hairs versiform, single hair-like hyphae or cystidia 53–97 µm long × 4–5 µm at the base, tapered to the tip, some with septa, scattered or in clusters, brittle, thin-walled, hyaline in KOH. Other hyphae present are claviform, erect, cylindrical or sinuate with an irregular thickness to the cell wall that resembles knobs or “spines”. Some globose or constricted hyphae emerging from isodiametric hyphae, 3–10 µm wide. Inner layer (subcutis) 50–225 µm thick, of prostrate and interwoven, hyphae gradually intermixing into gleba, hyaline in KOH and trypan blue, hyphae 2–5 µm wide. Some young specimens show noticeable prostrate cylindrical, claviform or vermiform hyphae along the subcutis that are thick-walled. Veins formed by hyaline, thin-walled, interwoven hyphae.

***Ascospores*** subglobose, globose to broadly ellipsoid, 23–58 × 18–48 µm without ornamentation, alveoli 2–7 µm tall, 7–10 alveolar meshes along the spore length, 5–6 across, polygonal (4–6 sides), cell wall 2–3 µm thick. 1-spored asci have spores that are 42–58 × 27–48 µm, 2-spored asci have spores that are 25–52 × 23–40 µm, 3-spored asci have spores that are 27–40 × 20–30 µm, 4-spored asci have spores that are 23–38 × 18–28 µm, 5-spored asci have spores that are 25–32 × 18–25 µm, yellowish to light brown in KOH and Melzer´s reagent. Asci globose, subglobose to broadly ellipsoid, without pedicel, 62–95 × 57–77 mm, hyaline in KOH, yellowish to brownish in Melzer´s reagent, thin-walled (immature asci thick-walled, up to 7.5 µm thick).

**Figure 2.**
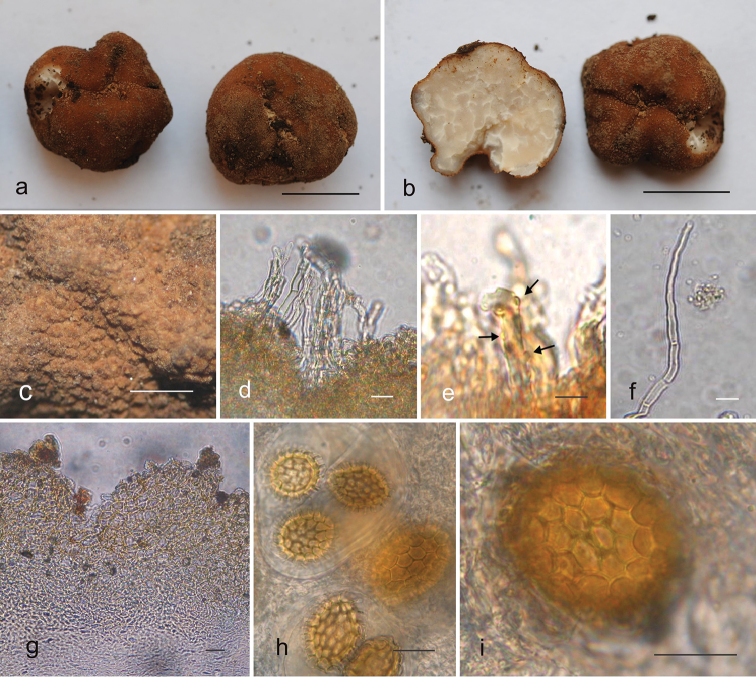
**a–i** *Tuber aztecorum* (holotype ITCV 993). a; Two ascomata showing the peridial surface (bar = 1 cm) **b** Ascoma in cross-section showing peridial surface and glebal surface (bar = 1 cm) **c** Peridial surface magnified showing the verrucose surface (bar = 1 mm) **d** Clusters of erect hyphae emanating from the peridial surface (bar =10 µm) **e** A single surface hair-like hypha (bar = 10 µm) **f** Cystidium (bar = 10 µm) **g** Cross section of peridium showing pseudoparenchyma-like epicutis (bar = 20 µm) **h** Ascospores within asci in surface view showing the alveoli (bar = 20 µm) **i** Ascospore within asci in surface view showing the alveoli magnified (bar = 20 µm).

##### Distribution and Ecology.

MEXICO, state of Mexico La Puerta, National Park Nevado de Toluca. Hypogeous, gregarious in volcanic rock soil in an *Abies religiosa* forest at 3065 m. N 19°11.662', W099°48.537'. 29 July 2010.

##### Additional collections examined.

Mexico, state of Mexico, La Puerta, National Park Nevado de Toluca, Guevara 1109 (paratype ITCV1109, GB KY271790), Guevara 1110 (paratype ITCV 1110), 29 July 2010.

## Discussion

Molecular data confirm that *T. aztecorum* belongs to the Maculatum clade, which is distinct from the Puberulum and Latisporum clades. *Tuber aztecorum* is morphologically and ecologically distinct from other known *Tuber* species (Fig. 1). *Tuber aztecorum* is a sister species to *T. castilloi*, however, *T. castilloi* differs by having larger spores, 27–63 × 20–40 µm, without irregular thickness to the cell wall and is associated mainly with *Quercus* spp. (Guevara et al. 2013a, b). Also *T. aztecorum* resembles *T. guevarai* but *T. guevarai* has narrower spores that are 18–55 × 16–42 µm (Guevara et al. 2013b). Although *T. aztecorum* belongs to the Maculatum clade, it is also morphologically similar to other *Tuber* species outside this group. It was preliminarily identified as *T. gibbosum* in the Gibbosum clade due to its association with *Abies religiosa* and the presence on the peridium of hyphae with irregular swellings in *T. aztecorum* (Guevara et al. 2013a, Bonito et al. 2010b). However, close morphological analysis and further molecular analysis revealed that it was not closely related to the Gibbosum clade. *Tuber aztecorum* is also similar to other species that belong to the Puberulum clade. *Tuber foetidum* is similar in its dark brown to reddish-brown peridium. However, *T. foetidum* has peridial cells 20–30 µm wide, lacks hairs, ascospores that are 25–44 × 21–32 µm and it grows under *Larix*, *Quercus* and *Fagus* (Jeandroz et al. 2008, Pegler et al. 1993). It is similar to *T. puberulum*, *T. rapaeodorum* and *T. borchii* from Europe. These three *Tuber* species have a dense and conspicuous fine epicutis. *Tuber puberulum* is frequently found in association with both *Fagus* and *Larix*. *Tuber rapaeodorum* has a paler ascoma surface with thinner cystidia, ellipsoid spores and is associated with *Quercus*, *Larix*, *Taxus*, *Pinus*, and *Fagus*. *Tuber borchii* has a pale, whitish or yellowish ascoma surface with abundant peridial hairs, 80% of its spores are ellipsoid and it is usually associated with *Fagus* or *Larix* (Pegler et al. 1993, Montecchi and Sarasini 2000, Halász et al. 2005, Wang et al. 2007, Jeandroz et al. 2008). *Tuber latisporum* differs morphologically from *T. aztecorum* by its conspicuously pubescent peridium, spores that are 24–49 (-51) × 20–40 (-44) µm and its association with *Pinus armandii* in China (Chen and Liu 2007).

In conclusion, morphological and sequence analysis of ITS rDNA can distinguish *T. aztecorum* from previously described species with strong bootstrap support and confidence (Halász et al. 2005, Wang et al. 2007, Jeandroz et al. 2008, Bonito et al. 2010a, b). The number of formally described *Tuber* species continues to grow (Table 1).

## Supplementary Material

XML Treatment for Tuber aztecorum
